# Co-precipitation molecules hemopexin and transferrin may be key molecules for fibrillogenesis in TTR V30M amyloidogenesis

**DOI:** 10.1007/s11248-017-0054-x

**Published:** 2017-12-29

**Authors:** Mika Ohta, Aki Sugano, Naoya Hatano, Hirotaka Sato, Hirofumi Shimada, Hitoshi Niwa, Toshiyuki Sakaeda, Hajime Tei, Yoshiyuki Sakaki, Ken-ichi Yamamura, Yutaka Takaoka

**Affiliations:** 1 Division of Medical Informatics and Bioinformatics, Kobe University Hospital, Kobe, 650-0017 Japan; 2 The Integrated Center for Mass Spectrometry, Kobe University Graduate School of Medicine, Kobe, 650-0017 Japan; 3 Department of Pathology, Division of Anatomical and Cellular Pathology, Iwate Medical University, Morioka, 028-3694 Japan; 4 Institute of Molecular Embryology and Genetics, Kumamoto University, Kumamoto, 860-0811 Japan; 5 Department of Phamacokinetics, Kyoto Pharmaceutical University, Kyoto, 607-8414 Japan; 6 Human Genome Center, Institute of Medical Science, The University of Tokyo, Tokyo, 108-8639 Japan; 7 Yamamura Project Laboratory, Institute of Resource Development and Analysis, Kumamoto University, Kumamoto, 860-0811 Japan

**Keywords:** Proteomic analysis, TTR amyloidogenesis, Hemopexin, Transferrin, Molecular dynamics simulation

## Abstract

The disease model of familial amyloidotic polyneuropathy—7.2-hMet30 mice—manifests amyloid deposition that consists of a human amyloidogenic mutant transthyretin (TTR) (TTR V30M). Our previous study found amyloid deposits in 14 of 27 7.2-hMet30 mice at 21–24 months of age. In addition, non-fibrillar TTR deposits were found in amyloid-negative 7.2hMet30 mice. These results suggested that TTR amyloidogenesis required not only mutant TTR but also an additional factor (or factors) as an etiologic molecule. To determine the differences in serum proteome in amyloid-positive and amyloid-negative mice in the 7.2-hMet30 model, we used proteomic analyses and studied serum samples obtained from these mice. Hemopexin (HPX) and transferrin (Tf) were detected in the serum samples from amyloid-positive mice and were also found in amyloid deposits via immunohistochemistry, but serum samples from amyloid-negative mice did not contain HPX and Tf. These two proteins were also not detected in non-fibrillar TTR deposits. In addition, in silico analyses suggested that HPX and Tf facilitate destabilization of TTR secondary structures and misfolding of TTR. These results suggest that HPX and Tf may be associated with TTR amyloidogenesis after fibrillogenesis in vivo.

## Introduction

In type I familial amyloidotic polyneuropathy (FAP), amyloid deposits are mainly composed of variant transthyretin (TTR) (Glenner 1980a, b). The most common amyloidogenic TTR is TTR V30M (Sakaki et al. 1989). Clinical symptoms of FAP result from amyloidosis occurring in peripheral nerves and essential organs such as the intestines, kidney, and heart, with the result being various syndromes that lead to death (Glenner 1980a, b). The age at onset differs widely and varies even in the same family. This finding suggests the presence of factors other than mutations in the TTR gene as causes of FAP.

Various approaches, such as animal models, have been used in attempts to understand this pathological process. We previously developed transgenic mouse models expressing TTR V30M that may allow us to investigate amyloidogenesis in vivo (Nagata et al. 1995; Takaoka et al. 1997, 2004; Wakasugi et al. 1987; Yamamura et al. 1987; Yi et al. 1991). These transgenic models provide an effective tool for studying the mechanisms of TTR amyloidogenesis. Our previous research with 7.2-hMet30 mice revealed amyloid deposits in 14 of 27 7.2-hMet30 mice at 21–24 months of age, as well as non-fibrillar TTR deposits in some Congo red-negative transgenic mice (Takaoka et al. 2004). On the basis of these data, we propose the following hypotheses: (1) the mis-folded monomer variant TTR forms aggregates to produce non-fibrillar deposits, and (2) amyloidogenic alterations occur as the result of another factor or factors.

The in silico approach based on structural biology has recently become a powerful tool for molecular pathological analysis (Nagai et al. 2017; Nakano et al. 2014; Ogasawara et al. 2016). We also used this method recently to investigate the effect of TTR prophylactic efficacy (Mu et al. 2015; Qiang et al. 2017). The process of TTR fibril formation was also analyzed by means of in silico analyses. Yang et al. (2003) showed substantial structural changes in amyloidogenic TTR L55P by using molecular dynamics (MD) simulations. Lei et al. (2004) reported that MD analysis found substantial local and global structural changes in TTR variants.

Animal models and in silico structural analyses are thus effective choices for pathological analysis of TTR amyloidosis. In this report, we describe differences in serum proteins in amyloid-positive 7.2-hMet30 mice maintained under conventional (CV) conditions and amyloid-negative 7.2-hMet30 mice maintained under specific-pathogen-free (SPF) and CV conditions. We also detail the relationships between proteomic differences and co-precipitates in amyloid deposits. In addition, we used in silico techniques to investigate new protein factors associated with TTR V30M amyloidogenesis. These results led us to a new hypothesis for the participation of new protein factors in TTR amyloid formation.

## Materials and methods

### Transgenic mice

In this study we used 7.2-hMet30 transgenic mice, which possessed 20–25 copies of the 7.2-kb fragment of the human TTR promoter with TTR cDNA in the cDNA expression vector, that were described previously (Takaoka et al. 2004). Mice were assigned to different housing groups: CV conditions or SPF conditions. All mice in the CV conditions group were kept under the same SPF conditions until the age of 8 months at the animal experimental facilities at Kumamoto University, when they were transferred to CV housing conditions at ARK Resource Co., Ltd. (the former Inoue Experimental Animal Center, Kumamoto, Japan) for the next 16 months. The 7.2-hMet30 mice kept under SPF conditions were kept under the same SPF conditions in the animal experimental facilities at Kumamoto University. All mice were 24 months of age when analyses were performed for this research.

All mice were treated according to the Standards Relating to the Care and Management, etc. of Experimental Animals (Ministry of the Environment, Tokyo, Japan). This study was performed in accordance with the approval of the Ethics Committee for Animal Experiments of Kumamoto University and the Institute of Medical Science, the University of Tokyo.

### Comprehensive analysis and western blotting of serum obtained from 7.2-hMet30 mice

Mouse serum samples were investigated by means of 12% sodium dodecyl sulfate-polyacrylamide gel electrophoresis (SDS-PAGE) and proteomic analysis. Each Coomassie Brilliant Blue-stained gel lane was cleaved into 1-mm lengths for in-gel trypsin digestion. The digested peptides were subjected to liquid chromatography (LC) and Q-Tof-2 quadrupole/time-of-flight (TOF) hybrid mass spectrometry (MS) (Micromass, Manchester, UK) according to a previously described protocol (Hatano and Hamada 2008). Differences in detected proteins obtained from amyloid-positive serum samples were identified. To verify protein differences found with MS/MS, we utilized the same antibodies in immunohistochemical and western blotting analyses with methyl alcohol-treated nylon membranes (Hybond N^+^; Amersham, Buckinghamshire, UK) and ECL Prime Western Blotting Detection Reagent (GE Healthcare UK Ltd, Buckinghamshire, England), as described previously (Takaoka et al. 2004).

### Histochemical and immunohistochemical analysis

Transgenic mice, under ether anesthesia, were treated at 24 months of age. Tissues were excised, fixed in 10% neutral formalin, and embedded in paraffin. For histochemical analysis of amyloid, tissue sections were stained with Congo red, and immunohistochemical analysis proceeded as described previously (Takaoka et al. 2004). Congo red-stained sections were examined by using a polarized microscope to detect the apple green birefringence emitted from the amyloid. For immunohistochemistry, the same paraffin sections were analyzed via rabbit anti-human TTR (MBL, Nagoya, Japan), anti-human hemopexin (HPX) rabbit polyclonal IgG (MBL, Nagoya, Japan), anti-mouse transferrin (Tf) rabbit polyclonal IgG (Inter-Cell Technologies, Hopewell, NJ, USA), and an avidin–biotin–peroxidase complex (ABC kit; Vector, Burlingame, CA, USA). To assess the specificity of the immunostaining, normal rabbit serum was substituted for the specific primary antibodies. As a negative control, the same procedure was used but without the primary antibodies, as described previously (Takaoka et al. 2004).

### In silico analysis of the effect of HPX and Tf bound with TTR V30M

The 3-D structures of wild-type human TTR (hTTR) and hTTR V30M dimer were obtained from the Protein Data Bank (PDB ID: 1F41 for wild-type hTTR, 1TTC for V30M dimer). The 3-D structure of the hTTR monomer (chain A) was “soaked” into water molecules and subjected to structural optimization by using GROMACS software (Abraham et al. 2015) with the AMBER03 force field, which was followed by a 250-ps heating process to reach a 310 K starting temperature. After heating, a 5000-ps production run was performed with the NPT ensemble. To determine the complex of the hTTR monomer with HPX or Tf, docking simulation was performed with ZDOCK software (Chen et al. 2003).

The 3-D structures of these proteins were downloaded from the PDB or ModBase (Pieper et al. 2011) as follows: mouse HPX (mHPX), ModBase model ID: f45bd5ef1540ddebb85169ea24254421; mouse Tf (mTf), ModBase model ID: 84e87ae66f1564521901f9b71f7cb525; human HPX (hHPX), ModBase model ID: 4c89fa1b9fd4d3df13dd69ae85ae65e9; human Tf (hTf), PDB ID: 3V83. Docking simulation was performed with all molecular surfaces defined as docking sites in each protein. After 2000 docking runs, binding models for hTTR V30M with HPX or Tf were determined by using the binding affinity (ZDOCK score).

We performed MD simulation analysis for each binding model: hTTR V30M with mHPX, mTf, hHPX, or hTf. Each structure was “soaked” into water molecules, and MD simulations were performed with GROMACS as described above. The secondary hTTR structures along the MD trajectories were analyzed by means of the DSSP program (Touw et al. 2015) according to previous research (Yang et al. 2003). The root-mean-square deviations (RMSDs) from the 5000-ps MD trajectory were analyzed by using the rmsdist GROMACS inbuilt tool.

### Statistical analysis

Statistical analyses were performed with R software (R Core Team 2015). Differences in ZDOCK scores were analyzed by using the unpaired Student’s *t* test (TTR and HPX, TTR and Tf). Data are presented as mean ± SD. A *p* value of < 0.05 was regarded as significant.

## Results

### SDS-PAGE and proteomic analyses of mouse serum

**Fig. 1:**
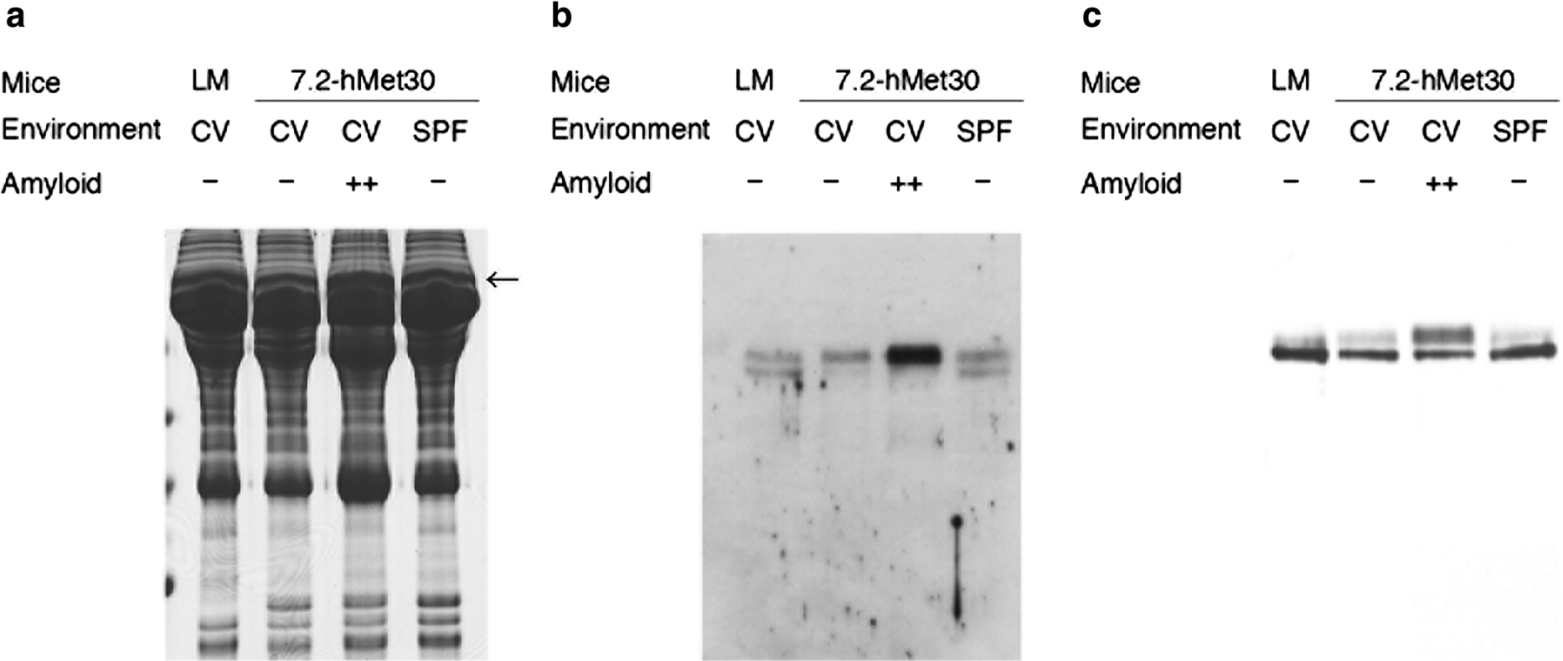
Comprehensive analysis of serum proteins in amyloid-positive and amyloid-negative 7.2-hMet30 mice. Differences in amyloid-positive serum were found in the band pointed by an arrow by means of proteomic analysis after SDS-PAGE. HPX and Tf were detected by MS analysis and they were found only in the amyloid positive serum in the band with an arrow (**a**). The presence of the protein differences detected, HPX (**b**) and Tf (**c**), was confirmed by using western blotting. LM, littermate

Our previous research demonstrated amyloid deposits in 6 of 19 24-month-old 7.2-hMet30 mice (Inoue et al. 2008), and non-fibrillar TTR deposits were found in 2 of 12 Congo red-negative transgenic mice (Takaoka et al. 2004). To determine the differences between amyloid-positive and amyloid-negative transgenic mice, we performed proteomic analyses of serum. In serum samples from mice subjected to SDS-PAGE, the protein band pattern was almost identical in amyloid-negative 7.2-hMet30 and control mice but not in amyloid-positive mice (Fig. 1a). After in-gel digestion, the different proteins in amyloid-positive serum identified by using LC–MS/MS analysis were as follows: mHPX, mTf, and mouse apolipoprotein A1. To verify the protein differences observed with MS/MS analysis, differences in HPX and Tf in amyloid-positive serum were confirmed by means of immunoblotting (Fig. 1b, c) without mouse apolipoprotein A1.

### Immunohistochemical analyses of TTR amyloid in 7.2-hMet30 mice

**Fig. 2:**
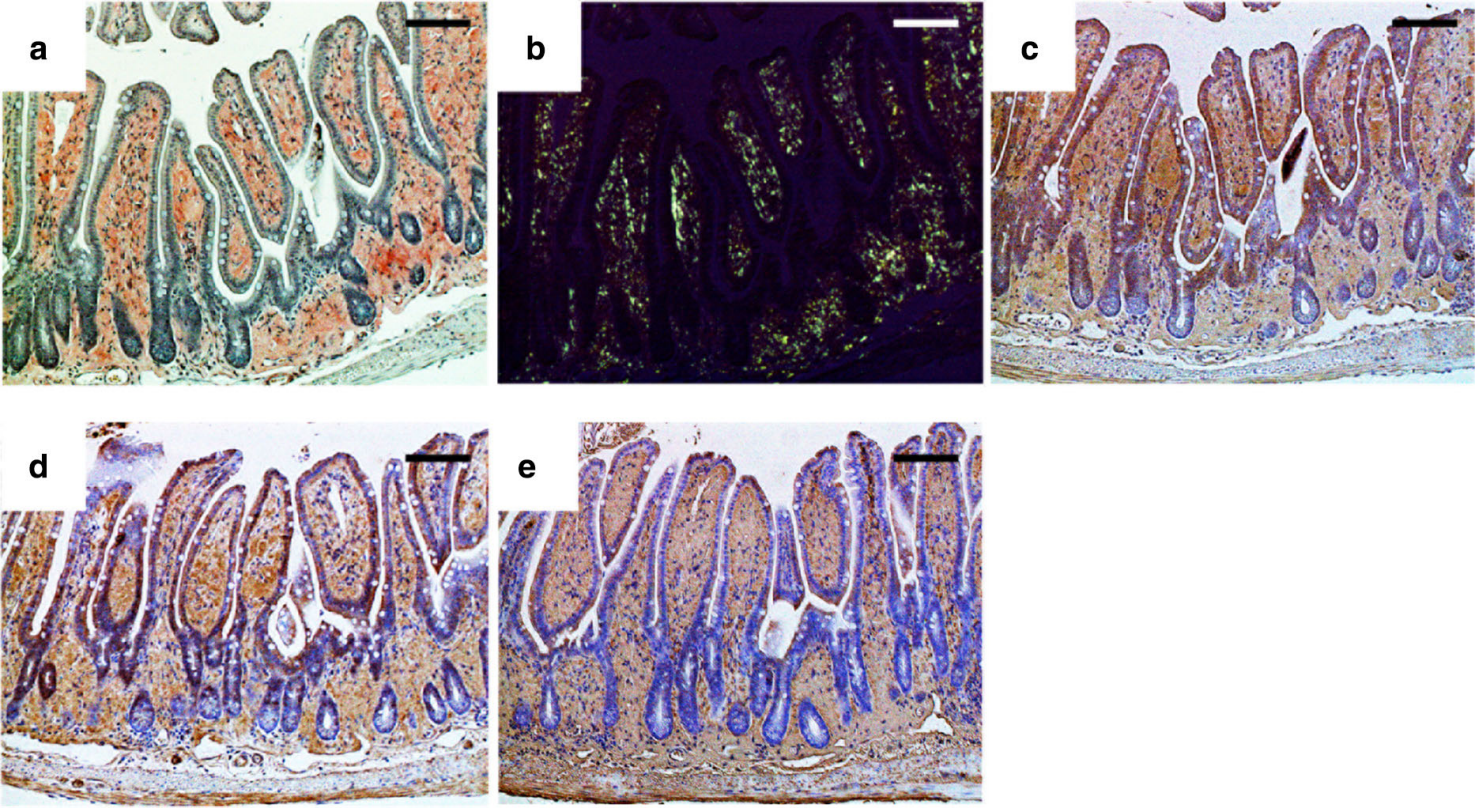
Histochemical and immunohistochemical analyses of amyloid deposits in the small intestine of a 7.2-hMet30 mouse kept under CV conditions. Congo red-positive deposits were present in the lamina propria of the small intestine (**a**). These deposits showed apple green birefringence under polarized light (**b**). Amyloid deposits demonstrated positive reactions with anti-human TTR antibody in our immunohistochemical analysis (**c**). Immunohistochemical analysis suggested co-precipitation of HPX in TTR amyloid deposits (**d**), and the presence of Tf (**e**). Bars = 100 μm. (Color figure online)

Immunohistochemical analysis was used to determine the co-precipitation of mHPX and mTf with TTR amyloid. Amyloid deposits were present in the lamina propria of the small intestine of 7.2-hMet30 mice kept under CV conditions (Fig. 2a, b). These amyloid deposits reacted with anti-hTTR antibody (Fig. 2c). This result demonstrates that these amyloid deposits were of hTTR V30M origin. HPX was detected in the same region as that with hTTR V30M (Fig. 2c, d). This finding suggests that mHPX was co-precipitated with TTR amyloid. In the Tf staining (Fig. 2e), antibody was reacted in the same region of amyloid deposits and also in small intestine muscularis propria. These results may suggest that mTf happened to precipitate in a whole small intestine, which means to exist with the TTR deposits in the lamina propria of small intestine mucosa and in the basal area of the lamina propria.

### In silico analyses of the effects of HPX and Tf on the conformation of TTR V30M

**Fig. 3:**
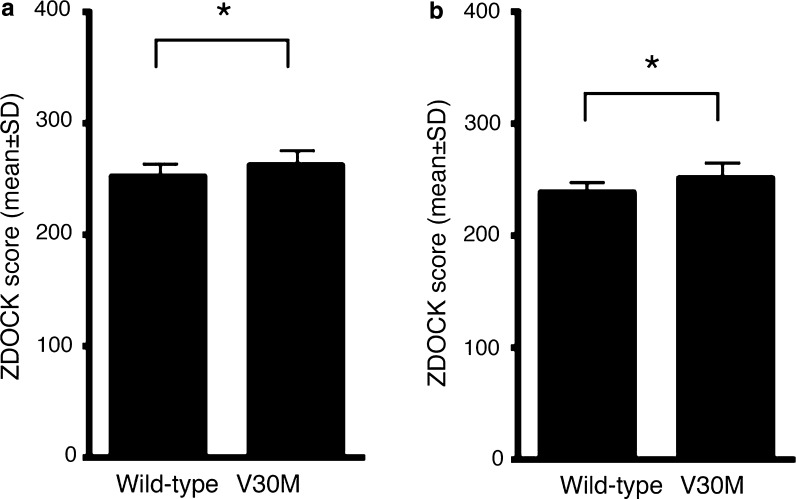
In silico binding stability of hTTR monomer with mHPX (**a**) and mTf (**b**). A high ZDOCK score means an energetically stable complex. *p < 0.001

**Fig. 4:**
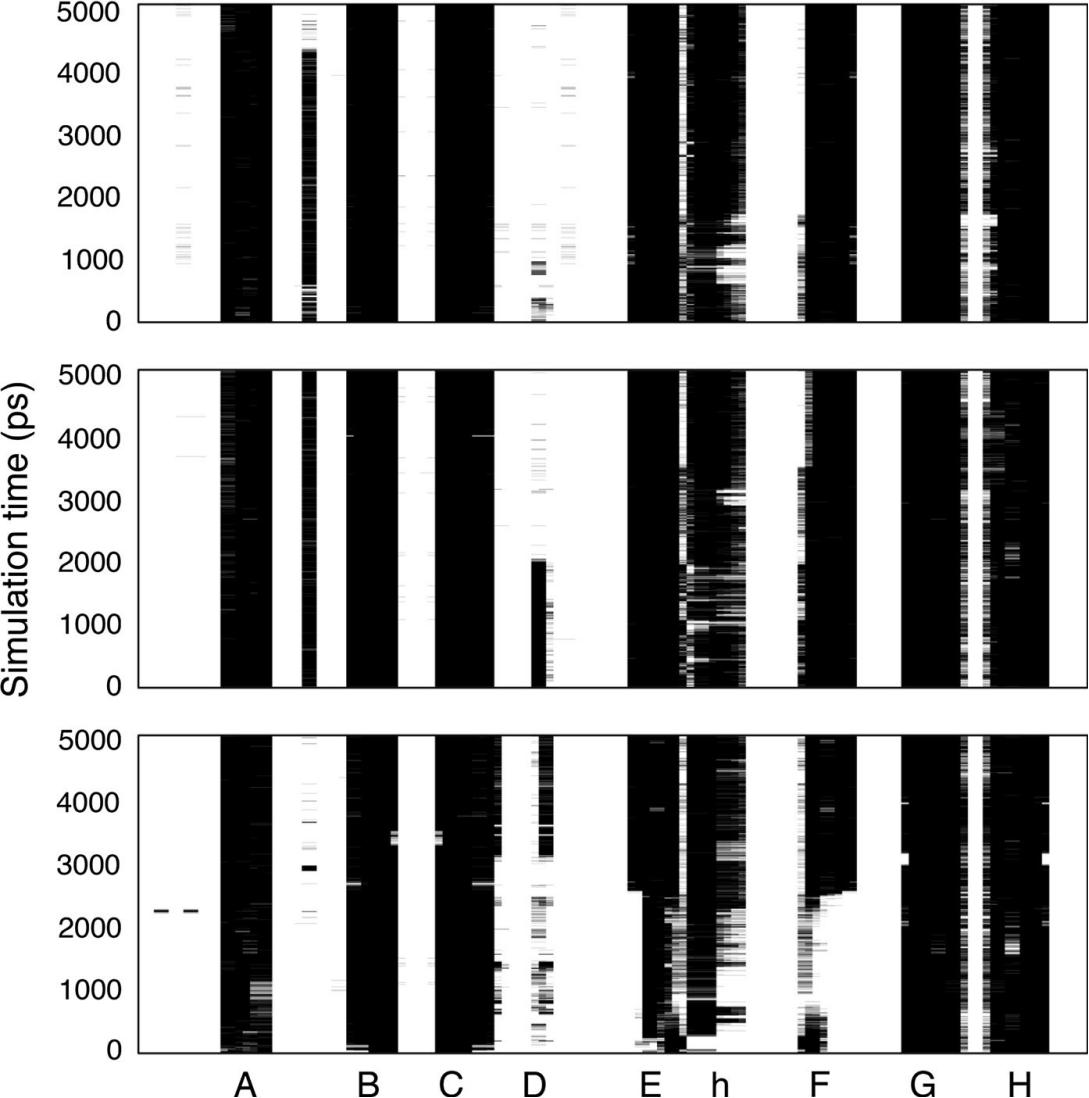
Analysis of the secondary structures along the MD trajectories for the hTTR V30M monomer (top), hTTR V30M monomer with mHPX (middle), and hTTR V30M monomer with mTf (bottom). The β-strands are named A to H. The h indicates the α-helix

**Fig. 5:**
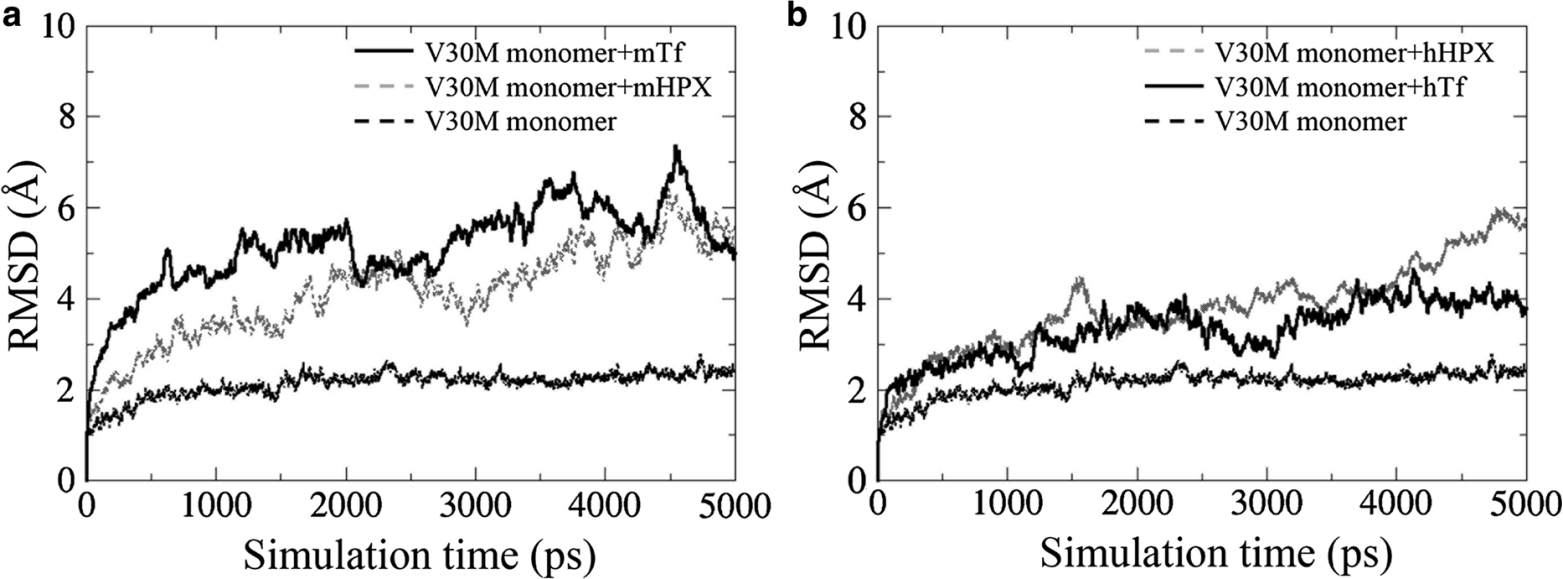
RMSDs for the 5000-ps MD trajectory of the main chain of the hTTR V30M monomer and it bound to mHPX or mTf (**a**) and hHPX or hTf (**b**)

To study the effects of mHPX and mTf on TTR amyloid formation, we performed in silico MD analyses. In the docking simulation of hTTR with mHPX or mTf protein, the binding stability of hTTR V30M with both mHPX and mTf was significantly higher than that of wild-type hTTR (Fig. 3). Analysis of the secondary structures via DSSP showed that the α-helix of hTTR V30M that bound to mHPX and that the α-helix and the β-strands of hTTR V30M that bound to mTf underwent substantial changes (Fig. 4). Certain common features of the secondary structural fluctuation existed for hTTR V30M: (1) the D strands in hTTR V30M seemed unstable in each simulation; and (2) the A, B, and C strands were relatively stable. These results are consistent with those of previous research (Yang et al. 2003). The average main-chain RMSDs between the MD simulation and the initial structure were as follows: 2.12 Å for the hTTR V30M monomer, 4.14 Å for the hTTR V30M monomer with mHPX, 5.13 Å for the V30M monomer with mTf, 3.78 Å for the hTTR V30M monomer with hHPX, and 3.30 Å for the hTTR V30M monomer with hTf (Fig. 5). These results suggest that the structure of hTTR V30M was greatly changed compared with the initial structure when hTTR V30M was bound to HPX or Tf.

## Discussion

In this study, we found that the proteins HPX and Tf that co-precipitated with amyloid manifested proteomic differences in amyloid-positive 7.2-hMet30 mouse serum. We also showed by means of in silico analyses that HPX and Tf may increase destabilization of the secondary structures of hTTR V30M and may thereby lead to amyloid fibrillogenesis.

We recently demonstrated that no amyloid deposition occurred in 7.2-hMet30 transgenic mice under SPF conditions; amyloid deposition occurred in 7.2-hMet30 only under CV conditions. In addition, we found that a long-term CV environment caused a large number of TTR amyloid deposits in 7.2-hMet30 mice (Inoue et al. 2008). These results suggested that another co-factor or other co-factors are needed for TTR amyloidogenesis. However, identifying these co-factors from among other environmental factors is difficult, because many factors including stress, physical and mental abuse, diet, and exposure to toxins, pathogens, radiation, chemicals, and weather exist.

To determine the effects of different environments, we used a comparative analysis of serum obtained from amyloid-positive and amyloid-negative 7.2-hMet30 transgenic mice. Differences in serum proteins existed only in amyloid-positive serum, as described in the following: HPX and Tf increased (Fig. 1b), which suggests that the manifestation of protein differences depends on the production of amyloid except under breeding environmental conditions, because the proteomic result for amyloid-negative 7.2-hMet30 mice under CV conditions was the same as that for 7.2-hMet30 mice under SPF conditions. However, these CV conditions for our 7.2-hMet30 mice (both amyloid-positive and amyloid-negative) were not entirely CV conditions, because all CV group mice were kept under SPF conditions until 8 months of age and then were kept under CV conditions for the remaining 16 months. In addition, the 24-month-old 7.2-hMet30 mice that were kept under complete CV conditions were 100% amyloid-positive (Inoue et al. 2008) and had the same serum proteome (data not shown). These data thus suggest that long-term CV conditions may cause these proteomic differences.

Immunohistochemical studies showed co-precipitation of mHPX and mTf in hTTR amyloid deposits in the small intestine of 7.2-hMet30 mice (Fig. 2). In addition, in silico studies showed that the binding affinities of mHPX and mTf with hTTR V30M were stronger than that with wild-type hTTR (Fig. 3). Indeed, no co-precipitation of HPX and Tf with Congo red-negative non-fibrillar TTR deposits occurred (data not shown). Also, the binding affinities of hHPX and hTf with hTTR V30M were stronger than that with wild-type hTTR (data not shown). These results suggest that HPX and Tf may be key molecules in TTR amyloid formation.

**Fig. 6:**
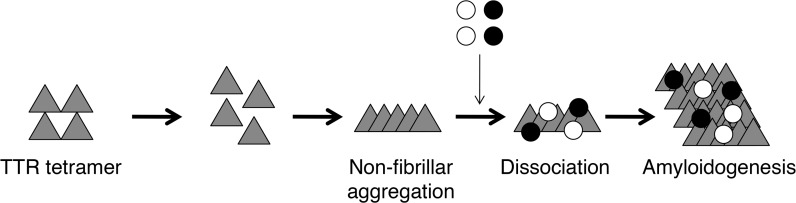
Schematic drawing of the enhancement of amyloidogenesis of hTTR V30M with co-precipitation of HPX or Tf. Triangles, TTR; white circles, HPX; black circles, Tf

Previously, Kelly (1998) showed that amyloidogenic TTR was more unstable than was wild-type TTR in vivo. Sekijima et al. (2005) reported that an amino acid substitution in TTR induced destabilization of native hTTR, which led to dissociation of the monomer and amyloidogenicity of this TTR V30M monomer. Certain in silico studies demonstrated the same structural changes in hTTR variants (Lei et al. 2004; Yang et al. 2003). To analyze the stability of hTTR V30M with co-precipitated mHPX or mTf, we performed MD analysis of hTTR V30M complexes with mHPX or mTf. Our MD analyses showed that the α-helix and the β-strands of hTTR V30M in these complexes evidenced substantial changes (Fig. 4) and that the structure of hTTR V30M with mHPX or mTf was greatly changed compared with the initial hTTR V30M structure (Fig. 5a). In addition, the same structural instability in hTTR V30M with hHPX and hTf was found in silico (Fig. 5b). These results and those of previous studies led us to propose the hypothesis that amyloidogenesis of TTR V30M was facilitated by HPX and/or Tf (Fig. 6). First, the TTR V30M tetramer dissociates into the native folded monomer (Colon and Kelly 1992; Lai et al. 1996), after which it forms aggregates so that non-fibrillar TTR deposits form (Takaoka et al. 2004). In this step, HPX and Tf did not co-precipitate with non-fibrillar hTTR deposits (data not shown). Next, HPX and/or Tf may bind to non-fibrillar TTR V30M (Fig. 3) and then increase destabilization of the secondary structures (Fig. 4) and misfolding (Fig. 5), thus leading to TTR amyloid formation.

We therefore demonstrated, by using the 7.2-hMet30 mouse model and in silico studies, that HPX and Tf may enhance TTR V30M amyloidogenesis. Our data also suggest that extracellular factors play an important role in TTR amyloidogenesis. Indeed, Bourgault et al. (2011) reported that extracellular factors such as sulfated glycosaminoglycans (e.g., heparin) facilitated monomeric TTR aggregation and induced tertiary and/or quaternary structural changes. Their results are consistent with our findings, and we thus propose that HPX and Tf are new candidate extracellular factors that can modulate TTR amyloid fibril formation. However, we analyzed tissues of only the 7.2-hMet30 model mice, so additional analysis of the co-precipitation of HPX and Tf with TTR amyloid in patients with FAP is warranted. And also, we will start further analysis for another target “contrapsin” which were lacking only in amyloid-positive serum by our MS analysis. Such investigations are in progress.
